# Aerobic Exercise Training Reduces Cannabis Craving and Use in Non-Treatment Seeking Cannabis-Dependent Adults

**DOI:** 10.1371/journal.pone.0017465

**Published:** 2011-03-08

**Authors:** Maciej S. Buchowski, Natalie N. Meade, Evonne Charboneau, Sohee Park, Mary S. Dietrich, Ronald L. Cowan, Peter R. Martin

**Affiliations:** 1 Energy Balance Laboratory, Division of Gastroenterology, Hepatology and Nutrition, Department of Medicine, Vanderbilt University, Nashville, Tennessee, United States of America; 2 Addiction Center and Psychiatric Neuroimaging Program, Department of Psychiatry, Vanderbilt University, Nashville, Tennessee, United States of America; 3 Department of Psychology, Vanderbilt University, Nashville, Tennessee, United States of America; 4 Department of Biostatistics, School of Medicine, Vanderbilt University, Nashville, Tennessee, United States of America; 5 Department of Radiology and Radiological Sciences, Vanderbilt University, Nashville, Tennessee, United States of America; 6 Vanderbilt University Institute of Imaging Sciences, Vanderbilt University, Nashville, Tennessee, United States of America; University of Granada, Spain

## Abstract

**Background:**

Cannabis dependence is a significant public health problem. Because there are no approved medications for this condition, treatment must rely on behavioral approaches empirically complemented by such lifestyle change as exercise.

**Aims:**

To examine the effects of moderate aerobic exercise on cannabis craving and use in cannabis dependent adults under normal living conditions.

**Design:**

Participants attended 10 supervised 30-min treadmill exercise sessions standardized using heart rate (HR) monitoring (60–70% HR reserve) over 2 weeks. Exercise sessions were conducted by exercise physiologists under medical oversight.

**Participants:**

Sedentary or minimally active non-treatment seeking cannabis-dependent adults (n = 12, age 25±3 years, 8 females) met criteria for primary cannabis dependence using the Substance Abuse module of the Structured Clinical Interview for DSM-IV (SCID).

**Measurements:**

Self-reported drug use was assessed for 1-week before, during, and 2-weeks after the study. Participants viewed visual cannabis cues before and after exercise in conjunction with assessment of subjective cannabis craving using the Marijuana Craving Questionnaire (MCQ-SF).

**Findings:**

Daily cannabis use within the run-in period was 5.9 joints per day (SD = 3.1, range 1.8–10.9). Average cannabis use levels within the exercise (2.8 joints, SD = 1.6, range 0.9–5.4) and follow-up (4.1 joints, SD = 2.5, range 1.1–9.5) periods were lower than during the run-in period (both P<.005). Average MCQ factor scores for the pre- and post-exercise craving assessments were reduced for compulsivity (P  = .006), emotionality (P  = .002), expectancy (P = .002), and purposefulness (P = .002).

**Conclusions:**

The findings of this pilot study warrant larger, adequately powered controlled trials to test the efficacy of prescribed moderate aerobic exercise as a component of cannabis dependence treatment. The neurobiological mechanisms that account for these beneficial effects on cannabis use may lead to understanding of the physical and emotional underpinnings of cannabis dependence and recovery from this disorder.

**Trial Registration:**

ClinicalTrials.gov NCT00838448]

## Introduction

Cannabis abuse or dependence and complications have increased in all age groups in the past decade in the United States. In 2009, approximately 16.7 million (6.6%) Americans age 12 or older reported cannabis use in the past month and 6.1 million used the drug on 20 or more days per month [1]. Treatment admissions primarily for cannabis dependence have also risen both in absolute numbers (∼1.2 million/year) and as a percentage of total addiction treatment admissions, from 7% in 1998 to 16% in 2009 [1]. Hence, there is a great public health need to develop safe and effective therapeutic interventions for cannabis use disorders.

Since there are no US Food and Drug Administration (FDA) -approved medications for cannabis dependence, treatment currently relies primarily on behavioral approaches generically used for treatment of all drug use disorders [2]. Aerobic exercise training, empirically part of a healthy drug-free lifestyle, has been considered as a promising behavioral approach in drug and alcohol treatment programs. Most extensively studied has been the effect of exercise on tobacco smoking. For example, a 2008 Cochrane Database reviewed 13 randomized trials in smokers or recent quitters, enrolled in an exercise program and followed for six months or more [3]. Certain of the reviewed studies showed significantly higher abstinence rates in a physically active group in comparison with a control group by the end of treatment [4]. However, to our knowledge, the direct effect of exercise on cannabis use *per se* has not been previously reported. In two very recent pilot studies, exercise training (2–6 months) was used as treatment to reduce drug use in general. In the first study of 38 drug abusers (51% of whom abused cannabis), the 58% who completed the program increased fitness level and improved quality of life [5]. At the end of the exercise program, 15 of the 20 drug abusers at least downgraded their drug intake. In a second study of 16 drug-dependent patients, those who participated in at least 8 of 12 weekly exercise sessions had significantly better substance use outcomes than those who did not [6].

Based on reports that aerobic exercise produces a host of psychological effects potentially associated with reductions in substance abuse [6], [7], [8], [9], we hypothesized that the exercise may generically alter reward circuits such that exercise takes the place of cannabis use to a significant degree because of training, thereby reducing cannabis use as exercise becomes more reinforcing. The purpose of this pilot study was to examine the effects of a supervised 2-week moderate exercise program on cannabis craving and use in non-treatment seeking cannabis dependent adults in their normal living environment.

## Materials and Methods

The protocol for this trial and the CONSORT checklist are available as supporting information; see Checklist S1 and Protocol S1.

### Participants

We recruited for the aerobic exercise training program 14 cannabis-dependent adults who met criteria for primary cannabis dependence according to the Substance Abuse module of the Structured Clinical Interview for DSM-IV (SCID-IV)[10], but who were not interested in reducing or quitting cannabis use or seeking treatment. A positive urine drug test for cannabis on the study day was an absolute criterion for participation. Participants were recruited using flyers and by word of mouth, signed an informed consent document approved by the Vanderbilt University Institutional Review Board, and were compensated for transportation and the study visits. The study was conducted between March and August 2010. This manuscript does not include results from the fMRI part of the protocol performed separately (see Protocol S1).

Exclusion criteria included presence of another Axis 1 DSM-IV diagnosis in the past 6 months, having a chronic medical or neurological illness, and having taken any psychotropic or vasoactive medications within 6 weeks of screen day. Also excluded were persons who smoked more than 10 cigarettes per day in the last year, and who had current dependence, as determined by the SCID, on any psychoactive substance other than nicotine and/or cannabinoids. Persons participating in an organized form of exercise or exercised more than 2 h per week [11] in the last month and persons having orthopedic or other problems precluding them from performing the exercise protocol were also excluded. Of those recruited, two (1 male, 1 female) did not complete the study; therefore, these results are reported on a final sample of 12 participants (age 24.8±2.9 years, 8 females).

### Ethics Statement

Written informed consent was obtained from all participants after they were given a complete description of the study. The Institutional Review Board of Vanderbilt University approved the protocol and consent procedure; see Consent Form S1 and Approval Letter S1.

### Study design

**Figure 1:**
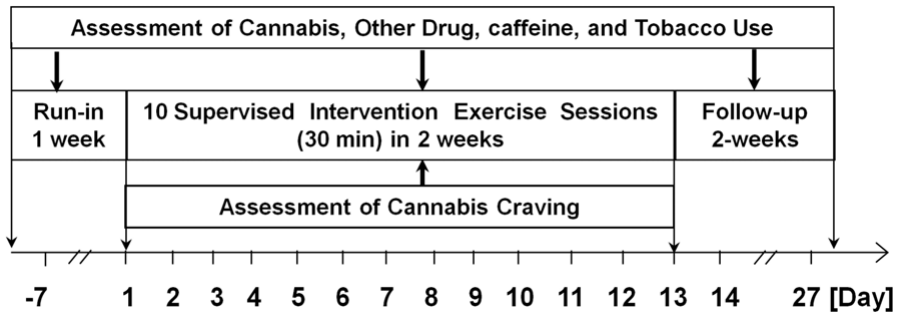
Experimental design. The study included Run-In (1 week), Exercise intervention (10 daily 30-minute treadmill sessions in ∼2 weeks), and Follow-Up (2 weeks) periods. Cannabis, other drug, caffeine and tobacco use were assessed from self-reports during all study periods. Cannabis craving was assessed pre- and-post each exercise session after viewing cannabis-related visual cues.

Sedentary or minimally active (<60 min/week of routine exercise) cannabis-dependent adults participated in this 2-week treadmill exercise program. After screening and a 7-day run-in period, participants came for 10 scheduled and supervised exercise intervention sessions (see Figure 1). On days with no exercise session scheduled, participants were encouraged to follow their daily routine and perform exercise in their normal living environment. Participants were able to make up for missed sessions with no more than 1 session per day. During the follow-up period (2 weeks), participants were asked to continue their daily routine without encouraging or discouraging them to continue exercise. Self-reported drug use was assessed for 1-week before, during, and 2-weeks after the exercise intervention.

At every session, before and after performing the exercise, each participant was presented with a set of visual cannabis cues on a computer monitor in conjunction with assessment of subjective cannabis craving determined using the Marijuana Craving Questionnaire (MCQ-SF) [12]. The cannabis -related cues included pictures of cannabis in different forms, people using cannabis, and paraphernalia. Three sets of cues were randomly assigned and were viewed during a 2-minute session performed in a separate semi-dark room.

#### Exercise protocol

Exercise was performed on a treadmill (Vision Fitness, Lake Mills, WI, US) at a target intensity of 60% of heart rate reserve that corresponds to approximately 60% of maximal aerobic capacity [13] for 30 minutes. Heart rate reserve, calculated as a difference between maximal and resting heart rate, was used as an indicator of intensity for exercise prescriptions [13] and monitored using an automatic monitor (DINAMAP® PRO, GE, St. Paul, MN, US). The intensity was adjusted to individual aerobic capacity and followed current guidelines that recommend limited thresholds of exertion level and time of exercise in sedentary populations [13]. All exercise sessions were conducted by the study exercise physiologists under medical oversight.

### Outcome Measures

#### Cannabis craving

Cannabis-related cues were selected from a variety of sources and altered using a visual graphics program to ensure clarity, brightness, color balance, and size. The stimulus set includes pictures of cannabis in different forms and its use (people smoking joints) and related paraphernalia (e.g. bongs, pipes). Craving was assessed after presentation of visual stimuli using the Marijuana Craving Questionnaire (MCQ-SF) [12]. The MCQ-SF is a Likert-based, 12-item self-assessment instrument for situational cannabis craving measurement with four factors (compulsivity, emotionality, expectancy, and purposefulness). The MCQ-SF validity to monitor the course of change in craving over time has been reported [12]. Each item is rated on a scale from 1 (strongly disagree) to 7 (strongly agree). Each of the factors is comprised of 3 items. Scores for each of the factors are derived by averaging the component item responses [14]. Internal consistency of the scores as measured by the Cronbach's alpha statistic were .86 (compulsivity), .93 (emotionality), .75 (expectancy), and .89 (purposefulness) scale in our study.

#### Comprehensive Drug Use Assessment

Lifetime drug use was assessed using a questionnaire that follows principles of Timeline Follow-back Method [15] and includes prompts for all major classes of drugs and includes assessments of age at onset, frequency of drug use, desired effects of drug or drug combination use [16], [17], [18], [19].

#### Current Drug Use

Participants received a calendar with instructions on how to use it to record drug use during the run-in, exercise intervention, and 2-week follow-up periods. Information was collected daily during the run-in period and every 5–7 days during the follow-up period. During the exercise intervention, the information was collected daily from the participants. The record included form (i.e. joints, blunts, bongs, chillums, bowls) and quantity of cannabis used, as well as use of any other drugs and alcohol. A trained interviewer reviewed the calendar with the participant using the Timeline Follow-back Method [15] to verify the information about drug use. Reported records were independently reviewed for reliability and correctness and entered into the statistical database.

#### Tobacco and caffeine use

Lifetime tobacco and caffeine use history was assessed using a validated questionnaire and current use was assessed daily by self-report [16], [17], [18], [19].

### Statistical analysis

Descriptive statistics were used to summarize the participant characteristics, as well as the variables of interest in this study. Because drug use history data were extremely skewed, median, minimum and maximum values are presented to summarize those distributions; otherwise mean ± SD are reported. Analysis of differences in overall cannabis use between each of the run-in, exercise, and follow-up periods were conducted using Wilcoxon Signed Ranks tests. Friedman tests were used to analyze changes in use within each of the periods. Wilcoxon Signed Ranks Tests were used to assess the statistical significance of changes in reported craving (MCQ Factor scores) from pre-to-post exercise. An alpha level of .05 was used for determination of statistical significance.

## Results

### Baseline Characteristics

Cannabis use history and current use patterns for the 12 participants are shown in Table 1. Regular cannabis use commenced at a median age of 14 years, which is slightly younger than the US average of 16.3 years (1), and the participants had regularly used cannabis a median of 8.5 years ranging from 1 to 15 years. Median current weekly cannabis use was 33.5 standard joints (min = 4, max = 140). Fifty percent of the participants also smoked cigarettes and all participants drank approximately 1 cup of coffee per day. Average time spent in moderate and vigorous physical activity [11], [13] was <60 min/week.

**Table 1:** Characteristics of cannabis use by study participants obtained from self-reports at baseline.

Drug Use History	Median(Min, Max)
Age at initial cannabis use (years)	14.0(12,18)
Age at regular cannabis use (years)	15.5(12,21)
Length of cannabis use (years)	8.5(1, 15)
Weekly cannabis use*	33.5(7,140)
Monthly cannabis use	90.0(14, 561)
Tobacco user	6 (50%)

*Major routes of cannabis administration were inhaling smoke from paper-wrapped joints or water pipe (e.g., bong or bowl), or consumed orally (cannabis added to baked brownies). Cannabis use was calculated in “standard joints”, where 1 joint equals 0.5 g of dry cannabis, 5 hits (deep inhalations) from a joint or a bowl, or 0.5 bowl.

### Protocol Adherence

The program goal of 10 planned exercise sessions was met by all of these participants over 15±2 day-period (min  = 12, max = 19). The mean intensity for all exercise sessions was within the moderate range (65–75% age-predicted heart rate).

### Cannabis Use

**Figure 2:**
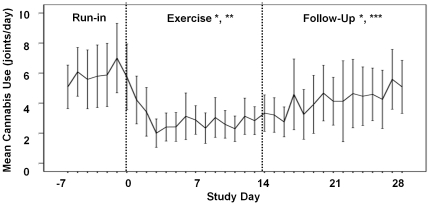
Cannabis use during the study. Mean (± 2 SEM) daily cannabis use (standard joints) during Run-In (1 week), Exercise Intervention (10 daily 30-minute treadmill sessions in ∼2 weeks), and Follow-Up (2 weeks). Cannabis use was calculated as “standard joints” where 1 joint equals 0.5 g of dry cannabis, 5 hits (deep inhalations) from a joint or a bowl, or 0.5 bowl. * - Decrease from Run-In period (P = 0.002). ** - Decrease in daily use from period onset (P = 0.006). *** - Increase in daily use from period onset (P = 0.003).

Daily cannabis use over the duration of the study is shown in Figure 2. Average use per day for all subjects during the run-in period was 5.9 joints per day (SD = 3.1) with a minimum average over the period of 1.8 and maximum average of 10.9 joints per day among subjects. During the exercise period, average use decreased to approximately 2.8 joints (SD = 1.6, min = 0.9, max = 5.4). Use increased on average during the follow-up period to 4.1 joints (SD = 2.5, min = 1.1, max = 9.5). Average cannabis use levels during the exercise and follow-up periods were statistically significantly lower than in the run-in period (both P = .002). There was a steady and statistically significant decrease in use per day during the exercise period (P = .006) with a corresponding statistically significant pattern of increase in use per day during the follow-up period (P = .003). While as noted, the average use within the follow-up period remained statistically significantly lower than the run-in period, use within the follow-up period was statistically significantly greater than during the exercise period (P = .010).

### Cannabis Craving

**Figure 3:**
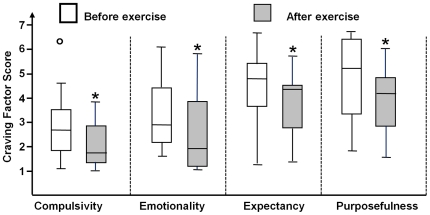
Cannabis craving during the exercise sessions. Box plots represent aggregated cannabis craving factor scores elicited by cannabis cues viewed before and after the standardized exercise sessions during Exercise Intervention (10 daily 30-minute treadmill sessions in ∼2 weeks). The cannabis craving factor scores were obtained from the cannabis-craving questionnaire (MCQ-SF) [12]. Changes in the cannabis craving factor scores from pre-to-post exercise were assessed using Wilcoxon Signed Ranks Tests. Each box represents middle 50% of results and horizontal line inside each box represents median for the score. *- Significant decrease from pre-exercise sessions (P<0.05).

Measures of cannabis craving are shown in Figure 3. Patterns of change over the exercise sessions within each of the pre- and post-exercise periods were not statistically significant. Therefore, those values were aggregated over the ten exercise sessions to generate average MCQ factor scores for the pre- and post-exercise craving assessments. Statistically significant reductions in average MCQ pre- to post-exercise factor scores were observed for compulsivity (P  = .006), emotionality (P  = .002), expectancy (P  = .002), and purposefulness (P = .002).

## Discussion

This study shows for the first time that participation in a supervised 2-week aerobic exercise program can reduce cannabis use in non-treatment seeking adults who meet criteria for cannabis dependence. Our findings also show that after exercise program completion, cannabis use significantly increased towards pre-treatment levels. Observed enduring effects were very likely caused by a relatively short (2 weeks) follow up and may well have returned to the baseline level with a longer period of observation. Consistent with the changes in cannabis use reported by participants, subjective cravings elicited by cannabis cues were also significantly reduced by exercise, suggesting the possibility that the potential therapeutic effect of exercise may be mediated via brain mechanisms responsible for cue-induced craving.

Why occasional self-administration of rewarding drugs can progress to out-of-control use or conversely, how some individuals engaged in such a self-destructive pattern of drug use may eventually stop taking drugs and recover from addiction have not been fully elucidated. However, learning-related processes, particularly in brain reward circuits, have been considered a partial explanation [20]. These same brain mechanisms have been invoked in behavioral addictions involving non-drug rewards, as is observed in overeating and obesity, problematic hypersexuality, and pathological gambling [21]. Analogously, it has been reported that exercise activates some of the same reward pathways as are activated by addictive drugs. For instance, acute bouts of exercise increase central dopamine concentrations [22], [23], [24], [25] and chronic exercise leads to sustained increases in dopamine concentrations and compensatory alterations in dopamine binding proteins in brain regions relevant to reward [26], [27], [28]. Moreover, aerobic exercise produces a host of beneficial effects, including improved self-esteem, weight control, and diminished depressive symptoms [29], [30], [31], [32], [33], [34].

It is not fully understood how exercise may affect drug use in general or cannabis use in particular because exercise is by no means universally rewarding. For instance, vigorous exercise intensities above the lactate threshold (a marker of aerobic exercise intensity) actually induce more negative affective responses than sub-threshold, self-selected exercise intensities[35]. It is hypothesized that this aversive exercise intensity threshold can be shifted to a higher level with training and hence may progressively make exercise more compelling even in those in whom it was initially aversive. Therefore, while it seems reasonable that rewarding effects of physical activity associated with exercise have the potential to supplant other behaviors such as drug use in the behavioral repertoire, this seems even more plausible over time with physical training.

A possible explanation for the beneficial effects of exercise on cannabis consumption may relate to affective changes associated with exercise that may alter cannabis use by diminishing depression and/or anxiety. For example, intermediary metabolism in muscle may be linked to affective changes as has been suggested by research on brain mechanisms of anxiety as triggered by lactate infusions [36]. An important question for future research is whether the beneficial effects of exercise on cannabis dependence are maintained over the long term as the present study was of relatively short duration. In addition, the neurobiological underpinnings of these beneficial effects on behavior may enhance our understanding of both cannabis dependence and recovery from this condition. Finally, the brain and peripheral mechanisms that account for these reductions in cannabis use may lead to valuable understanding of wellness, both physical and emotional.

Our study has several strengths. First, our study included cannabis users who were not interested in treatment, which suggests that this approach may be even more effective in individuals who are motivated to stop or limit cannabis use. Second, the study intervention was monitored and the treatment was adjusted to individual aerobic capacity eliminating a potential bias with exercise intensity and adherence.

Our study also has some limitations. First, we did not include a control condition but our goal was to assess the feasibility of conducting such studies and to determine any effects of this intervention on individual cannabis use. It is possible that other forms of intervention such as isometric exercise or body image methods will show effects similar to exercise as is the case in studies with tobacco (nicotine) dependence [37]. It has been shown that when smoked cannabis is mixed with tobacco, so that the consumption of cannabis is associated with tobacco smoking, cannabis use impedes the users' attempts to quit tobacco smoking [38], [39]. Second, our sample was relatively small. However, that we observed a highly significant effect even with these small numbers suggests an important effect size. Finally, we used self-report to assess cannabis use in a free-living environment with all its limitations. There is not a reliable and objective method to assess cannabis use in normal free-living environment since cannabinoid elimination from bodily fluids has such a long half-life. Consequently, most studies of cannabis consumption rely on self-reports. Nevertheless, self-report methods as we have employed in this study do provide reliable results, do not affect adherence, and are not burdensome to participants. The strategies we used to increase compliance (monetary incentives, follow-ups for missed appointments) while designed to increase validity, nevertheless reduced generalizability of our findings.

In summary, we found that participation in a supervised moderate exercise program could decrease cannabis use in association with reduced cannabis cue-induced craving in cannabis-dependent young adults who were not seeking treatment. Taken together, the findings of this pilot study suggest that a larger, adequately powered controlled trial is warranted to test the efficacy of moderate exercise as a component of treatment for cannabis dependence under real-world conditions. Such a study should also test the efficacy of a longer exercise program to determine the duration of treatment effects. Additionally, these studies should include a more diverse (age, ethnicity) population of individuals and especially cannabis dependent individuals who are motivated to seek abstinence from cannabis.

## Supporting Information

CONSORT Checklist S1 (DOC)

Protocol S1 (DOC)

Consent Form S1 (DOC)

Approval Letter S1 (PDF)

## References

[1] Substance Abuse and Mental Health Services AdministrationOffice of
Applied Studies NSH-A, editor (2010). Results from the 2009 National Surveys on Drug Use and Health:
National Finding..

[2] Budney A, Roffman R, Stephens R, Walker D (2007). Marijuana Dependence and its treatment.. Addiction Science and Clinical Practice.

[3] Ussher M, Taylor A, Faulkner G (2008). Exercise interventions for smoking cessation.. Cochrane Database Syst Rev.

[4] Van Rensburg K, Taylor A (2008). The effects of acute exercise on cognitive functioning and
cigarette cravings during temporary abstinence from smoking.. Human Psychopharmacology: Clinical and Experimental.

[5] Roessler K Exercise treatment for drug abuse - A Danish pilot study. Scand J
Public Health: Published ahead of print, June 21, 2010.

[6] Brown R, Abrantes A, Read J, Marcus B, Jakicic J (2010). A Pilot Study of Aerobic Exercise as an Adjunctive Treatment for
Drug Dependence.. Ment Health Phys Act.

[7] Brown R, Abrantes A, Read J, Marcus B, Jakicic J (2009). Aerobic exercise for alcohol recovery: rationale, program
description, and preliminary findings.. Behav Modif.

[8] Lynch WJ, Piehl KB, Acosta G, Peterson AB, Hemby SE (2010). Aerobic Exercise Attenuates Reinstatement of Cocaine-Seeking
Behavior and Associated Neuroadaptations in the Prefrontal
Cortex..

[9] Smith MA, Schmidt KT, Iordanou JC, Mustroph ML (2008). Aerobic exercise decreases the positive-reinforcing effects of
cocaine.. Drug and Alcohol Dependence.

[10] First M, Spitzer R, Gibbon M, Williams J (1997). Structured clinical Interview for DSM-IV Axis I Disorders,
Clinician Version (SCID-CV)..

[11] Matthews CE, Ainsworth BE, Hanby C, Pate RR, Addy C (2005). Development and Testing of a Short Physical Activity Recall
Questionnaire.. Medicine & Science in Sports & Exercise.

[12] Heishman SJ, Evans RJ, Singleton EG, Levin KH, Copersino ML (2009). Reliability and validity of a short form of the Marijuana Craving
Questionnaire.. Drug and Alcohol Dependence.

[13] American College of Sports Medicine (2008). ACSM's guidelines for exercise testing and prescription;
ACMS, editor..

[14] Heishman S, Singleton E (2006). Assessment of cannabis craving using the Marijuana Craving
Questionnaire.. Methods Mol Med.

[15] Sobell LC, Sobell MB (1992). Timeline follow-back: A technique for assessing self-reported
alcohol consumption. Measuring alcohol consumption: ippincott: Psychosocial
and biochemical methods:.

[16] Cowan R, Bolo N, Dietrich M, Haga E, Lukas S (2007). Occipital cortical proton MRS at 4 Tesla in human moderate MDMA
polydrug users.. Psychiatry Research: Neuroimaging.

[17] Cowan R, Haga E, Frederick B, Dietrich M, Vimal R (2006). MDMA use is associated with increased spatial BOLD fMRI visual
cortex activation in human MDMA users.. Pharmacology Biochemistry and Behavior.

[18] Cowan R, Wood J, Dietrich M, Frederick B, Lukas S (2008). Differential effects of amphetamine on red and blue light-induced
photic activation: A novel BOLD fMRI assay of human dopamine
function.. Synapse.

[19] Cowan RL, Joers JM, Dietrich MS (2009). N-acetylaspartate (NAA) correlates inversely with cannabis use in
a frontal language processing region of neocortex in MDMA (Ecstasy) polydrug
users: A 3 T magnetic resonance spectroscopy study.. Pharmacology Biochemistry and Behavior.

[20] Wise RA, Bozarth MA (1987). A Psychomotor Stimulant Theory of Addiction.. Psychological Review.

[21] Martin P, Petry N (2005). Are non-substance-related addictions really
addictions?. AM J Addictions.

[22] Fisher B, Petzinger G, Nixon K, Hogg E, Bremmer S (2004). Exercise-induced behavioral recovery and neuroplasticity in the
1-methyl-4-phenyl-1,2,3,6-tetrahydropyridine-lesioned mouse basal
ganglia.. Journal of Neuroscience Research.

[23] Hattori S, Naoi M, Nishino H (1994). Striatal dopamine turnover during treadmill running in the rat:
Relation to the speed of running.. Brain Research Bulletin.

[24] Meeusen R, Smolders I, Sarre S, de Meirleir K, Keizer H (1997). Endurance training effects on neurotransmitter release in rat
striatum: an in vivo microdialysis study.. Acta Physiol Scand.

[25] Petzinger GM, Walsh JP, Akopian G, Hogg E, Abernathy A (2007). Effects of Treadmill Exercise on Dopaminergic Transmission in the
1-Methyl-4-Phenyl-1,2,3,6-Tetrahydropyridine-Lesioned Mouse Model of Basal
Ganglia Injury.. J Neurosci.

[26] Gilliam PE, Spirduso WW, Martin TP, Walters TJ, Wilcox RE (1984). The effects of exercise training on [3H]-spiperone
binding in rat striatum.. Pharmacology Biochemistry and Behavior.

[27] MacRae P, Spirduso W, Walters T, Farrar R, Wilcox R (1987). Endurance training effects on striatal D2 dopamine receptor
binding and striatal dopamine metabolites in presenescent older
rats.. Psychopharmacology.

[28] Morgan (1982). Psychological effects of exercise.. Behav Med Update.

[29] Dunn AL, Trivedi MH, Kampert JB, Clark CG, Chambliss HO (2005). Exercise treatment for depression: Efficacy and dose
response.. American Journal of Preventive Medicine.

[30] Muller S, Dennis D, Gorrow T (2006). Emotional well-being of college students in health courses with
and without an exercise component.. Percept Mot Skills.

[31] Norris R, Carroll D, Cochrane R (1990). The effects of aerobic and anaerobic training on fitness, blood
pressure, and psychological stress and well-being.. Journal of Psychosomatic Research.

[32] Norris R, Carroll D, Cochrane R (1992). The effects of physical activity and exercise training on
psychological stress and well-being in an adolescent
population.. Journal of Psychosomatic Research.

[33] Veale D, Le Fevre K, Pantelis C, de Souza V, Mann A (1992). Aerobic exercise in the adjunctive treatment of depression: a
randomized controlled trial.. J Res Soc Med.

[34] Waade N (2004). Exercise improves self-esteem in children and young
people.. Aust J Physiother.

[35] Parfitt G, Rose EA, Burgess WM (2006). The psychological and physiological responses of sedentary
individuals to prescribed and preferred intensity exercise.. British Journal of Health Psychology.

[36] Johnson PL, Truitt WA, Fitz SD, Lowry CA, Shekhar A (2007). Neural Pathways Underlying Lactate-Induced Panic.. Neuropsychopharmacology.

[37] Ussher M, Cropley M, Playle S, Mohidin R, West R (2009). Effect of isometric exercise and body scanning on cigarette
cravings and withdrawal symptoms.. Addiction.

[38] Agrawal A, Madden PAF, Bucholz KK, Heath AC, Lynskey MT (2008). Transitions to regular smoking and to nicotine dependence in
women using cannabis.. Drug and Alcohol Dependence.

[39] Arpana A, Michael TL, Michele LP, Kathleen KB, Andrew CH (2008). Early cannabis use and DSM-IV nicotine dependence: a twin
study.. Addiction.

