# Supplementary material for: Aerobic Exercise Training Reduces Cannabis Craving and Use in Non-Treatment Seeking Cannabis-Dependent Adults
Source: PLoS One. 2011 Mar 8;6(3):e17465. doi: 10.1371/journal.pone.0017465 (PMC3050879; doi:10.1371/journal.pone.0017465)
Supplement: Protocol S1 — (DOC) [file pone.0017465.s002.doc]

# Interactions between Physical Activity and Cannabis Use in Adults

# (Pilot Study)

**Maciej Buchowski, Ph.D. -** Principal Investigator

Research Professor of Medicine and Pediatrics

Director, Energy Balance Laboratory

Department of Medicine

## Vanderbilt University Medical Center

**Peter Martin, M.D.,** Co- Investigator

Professor of Psychiatry

Director, Vanderbilt Addiction Center

Vanderbilt University Medical Center

## Ronald L. Cowan, M.D., Ph.D., Co- Investigator

## Assistant Professor of Psychiatry and Radiology and Radiological Sciences

Director, Psychiatric Neuroimaging Program

## Vanderbilt University Medical Center

**Sohee Park, Ph.D**., Co-Investigator

Associate Professor of Psychology and

Associate Professor, Center for Integrative Cognitive Neuroscience

Vanderbilt University Medical Center

## Evonne Charboneau, M.D., Co-Investigator

## Assistant Professor of Psychiatry

Department of Psychiatry

Vanderbilt University Medical Center

**Mary S. Dietrich, Ph.D**., Biostatistician - Co-Investigator

Assistant Professor of School of Nursing

Assistant Professor of Psychiatry

Vanderbilt University Medical Center

**John Gore, Ph.D**., Consultant

Professor of Radiology and Radiological Sciences

Professor of Molecular Physiology and Biophysics

Professor of Physics

Director, Vanderbilt University Institute of Imaging Science

Vanderbilt University Medical Center

**Table of Contents:**

**Study Schema**

1. **Background**
2. **Rationale and Specific Aims**
3. **Animal Studies and Previous Human Studies**
4. **Inclusion/Exclusion Criteria**
5. **Enrollment/Randomization**
6. **Study Procedures**
7. **Risks of Investigational Agents/Devices (side effects)**
8. **Reporting of Adverse Events or Unanticipated Problems involving Risk to Participants or Others**
9. **Study Withdrawal/Discontinuation**
10. **Statistical Considerations**
11. **Privacy/Confidentiality Issues**
12. **Follow-up and Record Retention**

**References**

**Appendices**

**Appendix A MRI Safety Screening Form**

**Appendix B Telephone Screening Script**

**Appendix C Drug Use Screening Form (applies to cases only)**

**Appendix D Mode Disorders Form (MINI) (applies to cases only)**

**Appendix E Cannibis Craving Questionnaire**

**Appendix F Food Craving Questionnaire**

**Appendix G Food Cues (CD)**

**Appendix H Cannabis Cues (CD)**

**Appendix G Recruitment Flyers**

**1.0. Background**

**Overview**

Growing evidence linking physical activity to a broad range of desirable health outcomes (e.g., cardio-respiratory health, musculoskeletal health, functional health, energy balance, cancer prevention) indicates the promise of exercise for promoting health and preventing chronic disease. In addition, basic physical activity research in animals and humans has demonstrated the influence of physical activity on the development of cognitive skills, including decision-making, social-emotional learning, executive control processes and attention and much has been learned from animal research about the neurobiological changes induced by exercise regimes. Moreover, intervention research on mood and physical activity indicates significant benefits on mood regulation, stress, and internalizing disorders, especially among depressed adults and children. These areas of research provide a starting point for understanding the role of physical activity across the lifespan in promoting general physical and mental health while simultaneously addressing chronic disease, psychiatric disorder, and substance use. However, few controlled experimental studies have been conducted to examine beneficial effects of exercise on cognition and other health-related behavioral or neurobiological processes and our understanding of the mechanisms that underlie these effects are limited.

This developmental proposal combines expertise from multiple disciplines to bring current knowledge and technological capabilities to the important public health problem of addiction. The overall goal is to explore the relationship between physical activity and addiction. The research team brings experience from addiction (Dr. Peter Martin), physical activity (Dr. Mac Buchowski), psychiatric neuroimaging (Drs. Ronald Cowan and Evonne Charboneau), experimental psychology (Dr. Sohee Park), biostatistics (Dr. Mary Dietrich); and neuroimaging physics (Dr. John Gore). The PI’s laboratory has a primary focus on physical activity and its role in health and disease.

The goal for this project is to obtain pilot data to support R01 application (RFA-DA-09-013; Interactions between Physical Activity and Drug Abuse) (DHHS 2008)for funding for a larger-scale study on interaction between physical activity and functional brain activity in cannabis addiction. Longer-range goals include research designed to improve the translation of existing knowledge of the effects of exercise, physical activity and inactivity into strategies for preventing and treating drug abuse and addiction. Another long-term goal is to research interactions between physical activity and over-eating as contributors to obesity and its health consequences.

**Physical Activity and Addiction**

There are important gaps in the current literature on physical activity and substance use. First, little research has addressed the broad array of substances of abuse; most has focused on tobacco cessation. Second, in the areas of substance use prevention and treatment, the science is not well-developed enough to associate physical activity with reduced risks of substance abuse for non-users and reduced use among those who are diagnosable with a drug use disorder.. Our understanding of how the changes in motivational, emotional, and cognitive processes induced by physical activity have functional significance for drug abuse and addiction is limited. Recent advances in neuroimaging technologies such as fMRI, MRI, PET and evoked response potentials for studying the human brain have the potential to define the effects of physical activity on the brain processes underlying drug abuse behavior and addiction. New animal models are being utilized to study the genetic, molecular, and neurobiological systems that underlie the changes at the cellular level that are triggered by physical activity that have functional consequences for behavior. The results are promising in that these techniques are capable of showing changes in brain structure and function that are related to physical activity. However, further research is needed to better understand the neurobiological and behavioral mechanisms that underlie the link between physical activity and improved psychological well-being.

Many of the brain structures and neural circuits affected by physical activity have also been implicated in the initiation of drugs of abuse, as well as the development of addiction. Given that physical activity has significant effects on some of these same neural networks, exercise may provide a prophylactic effect and/or an ameliorative treatment component in management of drug use disorders. Among the areas of gaps in knowledge are neurobiological and behavioral mechanisms responsible for the effects of physical activity on brain function, drug abuse behavior and addiction across the lifespan as well as research designed to improve the translation of existing knowledge of the effects of exercise and physical activity into strategies for the prevention and treatment of drug abuse.

**Physical Activity and Addiction Treatment**

Exercise has been shown to promote psychological well-being and is sometimes prescribed as an adjunct to treatment for psychiatric disorders such as anxiety and depression. Although recreation therapy is tacitly assumed to be complementary to pharmacological and psychosocial interventions in addiction treatment, relatively little mechanistic research has been conducted to understand the effects of exercise in the treatment of drug dependent individuals. A number of studies have examined the impact of exercise on abstinence rates among nicotine addicted individuals. Some of the findings have been promising, yet more rigorous research is needed that takes into account sample size, acceptability of exercise regimen, follow-up, and other methodological factors. Moreover, the effects of exercise on illicit drug dependence treatment is poorly understood and thus greatly needed.

**Reward System Activated Both by Natural and Drug-Induced Rewards**

Rewarding effects associated with their use provide the initial driving force to take drugs. Numerous studies have described acute effects of different rewarding drugs and the common denominator of their mode of action appears to be release of dopamine in nucleus accumbens.(Di Chiara and Imperato 1988). Similarly, natural behaviors that are essential in daily life and survival of the species also mediate release of dopamine in nucleus accumbens. Also other common behaviors such as playing computer games (Koepp, Gunn et al. 1998), listening to pleasant music (Blood and Zatorre 2001), or watching attractive faces (Aharon, Etcoff et al. 2001) mediate release of dopamine or increase energy consumption in nucleus accumbens. Non-drug associated behaviors can also develop into pathological behaviors such as anorexia nervosa, bulimia nervosa and pathological gambling (see 307.1, 307.51 and 312.31, respectively in Diagnostic and Statistical Manual 4:th edition, (DSM IV)). Similarly, excessive physical training can result in fatigue and mood disturbances as has been reported in overtrained humans (Armstrong and VanHeest). These symptoms are similar to hallmarks of withdrawal in substance abusers, mood disturbances such as depression and anxiety. Also, after withdrawal from an excessive natural rewarding behavior such as running or from addictive drugs the individual will show signs of depression and anxiety or other indications of negative affective states (Koob and Moal 1997). It is not known why long-term use of rewarding drugs can lead from controlled recreational use to out-of-control consumption. Such transition from controlled to compulsive reward seeking behavior is also seen with non-drug induced rewards. With daily repeated administration of the drug this effect gradually increases, a phenomenon termed behavioral sensitization (Wise and Bozarth 1987).

**Cannabis’ Effects on the Brain**

| Brain Region | Functions Associated With Region |
| --- | --- |
| Brain regions in which cannabinoid receptors are abundant | |
| Hippocampus | Learning and memory |
| Cerebellum | Body movement coordination |
| Cerebral cortex, especially cingulate, frontal, and parietal regions | Higher cognitive functions |
| Nucleus Accumbens | Reward |
| Basal ganglia   - Substantia nigra pars reticulata - Entopeduncular nucleus - Globus pallidus - Putamen | Movement Control |
| Brain regions in which cannabinoid receptors are moderately concentrated | |
| Hypothalamus | Body housekeeping functions (body temperature regulation, salt and water balance, reproductive function) |
| Amygdala | Emotional response, fear |
| Spinal cord | Peripheral sensation, including pain |
| Brain stem | Sleep and arousal, temperature regulation, motor control |
| Central gray | Analgesia |
| Nucleus of the solitary tract | Visceral sensation, nausea and vomiting |

**Health Consequences of Cannabis Abuse**

Acute (present during intoxication)

- Impairs short-term memory
- Impairs attention, judgment, and other cognitive functions
- Impairs coordination and balance
- Increases heart rate

Persistent (lasting longer than intoxication, but may not be permanent)

- Impairs memory and learning skills

Long-term (cumulative, potentially permanent effects of chronic abuse)

- Can lead to addiction
- Increases risk of chronic cough, bronchitis, and emphysema
- Increases risk of cancer of the head, neck, and lungs

**Regions of interest**

Exposure to cannabis, both acutely and chronically, is associated with changes in brain activity, many paralleling those found with other substance use disorders. These findings predominantly involve frontal, limbic and also cerebellar regions, with generally increased activity during exposure and generally decreased activity during abstinence. Findings seen during abstinence may persist even though they are not necessarily correlated with neuropsychological test results. Regions of differential brain activity appear to involve those implicated with other substance use disorders involving the extended dopamine reward pathways, but also a potential frontocerebellar network.

**2.0 Rationale and Specific Aims**

It is unknown how the response of cannabis users might compare to those of non-users in response to similar dose of physical activity. We will therefore compare brain activation during craving to cannabis, a natural reward (food) and a neutral scene (nature images). The food cues will allow us to determine if effects of exercise on brain activation and craving generalize to two different reward conditions and the nature scenes provide a neutral, no rewarding control. In addition to the analysis of predetermined regions of interest, whole-brain exploratory analyses will also be conducted to examine for additional brain regions showing associations between various conditions of interest and regional brain activation.

**Specific Aims**

1. To explore methodological factors involved in finding the relationship between PA and cannabis use.
   1. to examine the effects of cannabis and food cues on brain activation and craving in cannabis users and nonusers
   2. to examine the effects of vigorous exercise on brain activation and craving in response to cannabis and food cues.

Our working hypothesis is that the regional brain activation in response to drug cues will be different in cannabis users than in controls and that exercise will significantly alter brain responses to these cues in both groups. Our secondary hypothesis is that exercise will tend to normalize the abnormal brain activation observed in cannabis users.

Our expectation is that exercise will alter cue-responding in terms of brain activation or craving. It is possible that exercise may be a useful treatment in cannabis dependence. Future studies may examine this specific relationship.

There are no reports available exploring the relationship between physical activity and cannabis use.

Current data suggest that there are convergent findings regarding the chronic and acute effects of cannabis on brain activity

1. **Inclusion/Exclusion Criteria**

**Inclusion Criteria:**

Cases and Controls must:

- Be able to understand the study and provide written informed consent.
- Be male or female 18 -35 years of age.
- Be right handed
- Be in generally good health based on history, physical examination, electrocardiogram and laboratory findings.
- If female of childbearing potential, have a negative serum pregnancy test on study day

Cases must:

- Meet criteria for cannabis dependence as primary diagnosis as determined by the Substance Abuse module of SCID (Structured Clinical Interview for DSM-IV).
- Not currently seeking treatment for cannabis dependence.
- Have a positive urine drug test for cannabis on the study day
- Avoid alcohol and other recreational drug use (except cannabis and/or nicotine) for 48 h before testing.

**Exclusion Criteria:**

Cases and Controls must not:

- Be pregnant or lactating
- Have implanted electrical medical device (e.g. pacemaker, vagal nerve stimulator)
- Have non-secure metallic foreign bodies
- Have met criteria for another axis 1 diagnosis in the past 6 months
- Receive psychotropically active or vasoactive medications (within 6 weeks of screen day)
- Have chronic medical illness
- Have epilepsy
- Have a history of head injury that required hospitalization
- Have claustrophobia
- Have orthopedic or other problems precluding them from performing exercise protocol
- Have body mass index (BMI) less than 19 kg/m^2^
- Be heavy smokers (more than 10 cigarettes per day in last 5 years)
- Weighing more than 275 pounds (MRI table limit)

Cases must not:

- Have current dependence, as determined by the SCID, on any psychoactive substance other than nicotine and/or cannabinoid
- Have any serious medical or psychiatric illnesses and/or clinically significant symptoms, which in the judgment of the PI or his/her designee would make them unsafe, or would make compliance with the study protocol difficult or put the study staff at undue risk
- Be under court mandate to obtain treatment

Rationale for inclusion/exclusion criteria:

Participants will include otherwise healthy male and female (18 to 35 years) either cannabis dependence or controls who do not meet criteria for a cannabis abuse or dependence.

Participants in the two groups will be approximately matched by gender, age, height, and educational level. Only right-handed participants will be enrolled to minimize laterality variations in brain function. Overweight volunteers whose weight is greater than 275 pounds (within what we consider a practical safety margin to avoid exceeding the maximum recommended weight for the MRI scanner table of 300 pounds) will be excluded.

All participants will be screened for Axis I psychiatric pathology using a clinical interview conducted by or reviewed with Drs. Charboneau and Cowan to assess for the presence of medical and psychiatric pathology, respectively. Because the presence of an Axis I disorder, as reviewed above, is often co-morbid with cannabis dependence but might confound the interpretation of brain responses, Axis I conditions will be exclusionary for this pilot study.

Screening and study instruments:

- MRI safety screening form
- MINI-structured psychiatric interview (cases only)
- Drug use questionnaire (cases only)
- Nicotine and Caffeine questionnaire
- Marijuana Craving Inventory
- Food Craving Scale

**Screening and study instruments are attached as Appendices A-G.**

1. **Enrollment/Randomization**

Participant recruitment will be conducted using methods previously successful for the recruitment of cannabis users at Vanderbilt. These include flyers and advertisements in local print and audio media outlets, and mass email solicitation. The Vanderbilt Addiction Center will also provide recruitment support services, including listing the study on their Study Finder web site and talking about the study at public meetings. All advertisements will be IRB approved and indicate the research nature of the study. Participants will be screened by phone, and those meeting criteria for enrollment will be scheduled for the study. The study is not blinded (except during data analysis phases, when researchers are blind to exercise condition and group status) and the exercise intervention is randomized.

Written informed consent forms describing the experimental protocols and known risks and hazards, and approved by the Vanderbilt University Medical Center IRB, will be given to participants and the participants will be provided with a copy of the forms. Participants will have the opportunity to ask clarifying questions about the procedure prior to signing the consent forms. The consent will be signed under conditions that give free power of choice without intervention or undue duress, persuasion, coercion, or deception. Consent forms will be stored in a file cabinet in a locked office. Participants will be instructed that they are free to end the study at any time.

**Study Advertisements are attached as Appendix E-F.**  Font size, placement, and bolding will vary. Compensation sentence will not be larger or bolder than other text. Tear-off phone number may be added at bottom of flyers.

**Script for telephone screening is attached as Appendix B.**

**Informed consent form for patients and controls is included in IRB application.**

1. **Study Procedures**

We plan to enroll 35 participants. The study components will consist of an initial phone screen with the participant to verify provisional inclusion criteria. For applicants meeting provisional inclusion criteria, a screening visit will be scheduled for Informed Consent, explanation of study procedures, screening with study instruments and interviews, and an opportunity for a practice session in the mock MRI scanner. For applicants meeting inclusion criteria following screening, a study visit will be scheduled, preferably within 2 weeks of the screening day.

**Table 1. Visit Schedule**

| **Procedure***** | **Visit 1**  **(day 1)** | **Visit 2**  **(day 2-8)** | **Visit 3**  **(days 5-14)** | **Visit 4-12**  **(days 2-10)** |
| --- | --- | --- | --- | --- |
| Consent | X |  |  |  |
| Surveys/questionnaires***** | X | X | X | X |
| Fitness test* | X | X | X |  |
| EKG | X |  |  |  |
| Vital Signs | X |  |  |  |
| Exercise**,*** |  | X | X | X |
| fMRI**** | X | X | X |  |
| Urine Tests | X | X | X | X |
| Buccal cells or Saliva (DNA) | X |  |  |  |
| Breath test | X | X | X |  |

*VO2max – assessment of physical fitness. This test will be done during Visit #1, #2, or #3.

** exercise in randomized order. The participant will be asked to run or jog on a treadmill at a speed approximately 20-50% lower than your fast running speed and/or walk at a comfortable speed during Visit #4 – Visit #12.

*** - Participant may be asked to come to one, two, three, four, five, or six visits.

**** - functional magnetic resonance imaging (test to picture parts of brain). This will be done twice, either during Visit #1 and Visit #3 or during Visit # 2 and Visit #3.

*****On the days a subject is not scheduled for a visit, we may ask questions from the Marijuana Craving Questionnaire, Caffeine and Nicotine Use questionnaire, and Drug use (question Page 13 Question 1: Marijuana (as a dried plant) over the phone. The subject will get copies of these questionnaires so they will know questions we may ask them.

**Visit 1.**

Consent/Screening

After signing an informed consent, participants will be screened for psychiatric conditions using the MINI questionnaire. No included participants will have a lifetime Axis I psychiatric diagnosis other than cannabis or nicotine dependence for cases and nicotine dependence for controls.

Participants will also complete detailed substance abuse questionnaire using a time-line follow back method (Fals-Stewart et al. 2000).  The questionnaires probe drug use history in alcohol, cannabis, stimulants, hallucinogens, opiates, sedatives, dissociative anesthetics, anabolic steroids, and inhalants. The questionnaire contains items to indicate when a specific drug was last used, number of total lifetime episodes, episodes in the last monthand/or last week, whether the participant intended to use the drug in the future, and approximate amount of the drug used (Cowan et al. 2006; Cowan et al. 2007).

Participants will also complete a Spielberger Strait Trait Questionnaire which is a self-evaluation questionnaire that asks the participant how he or she feels at the moment and in general. They will also fill out a BIS/BAS questionnaire which asks questions about mood, emotions, and behaviors.

Urine pregnancy test – will be screened at each visit to rule out pregnancy (urine; QuPid One Step Pregnancy Test; Stanbio Laboratory, Inc. San Antonio, TX),

Recent drug use will be screened at each visit.

- urine; amphetamines, methamphetamines, barbiturates, benzodiazepines, cannabinoids, cocaine, opiates, PCP and tricyclic antidepressants; Triage Drugs of Abuse Panel, Biosite Diagnostics, San Diego, CA)
- breath - alcohol use (Alco Sensor III, Intoximeters, St. Louis, MO).

**VO_2_ max testing:** Prior to the test, supine and standing EKG’s will be performed to rule out any rhythmic abnormalities. We had a rough time when some of our normal controls came back with minor abnormalities and we had stated any abnormalities were exclusionary. Participants will be asked to perform a walk/jog test on a treadmill (Bruce Protocol) while oxygen intake and carbon dioxide production are measured.

Participants having a positive urine (other than cannabis for cannabis users), pregnancy, or alcohol screen will be removed from the study prior to MRI scanning.

**Mock Scanner Practice Session**

Prior to the fMRI scan day (preferably during the screening visit), screened and consented participants will be given an opportunity to visit the Imaging Institute and participate in a mock scanner session rehearsal to minimize anxiety and novelty effects. Participants will view neutral cues (using a different set of neutral cues than employed on the study day) while lying on a scanner table with audio playback of previously recorded scanner sounds. Participants may repeat the mock scanner rehearsal sessions until they are comfortable with the overall environment. Participants having intolerable anxiety or other difficulties that will likely interfere with successful study participation will be excluded at this point.

### Fasting

All participants will be asked to avoid the ingestion of any energy (calorie) or caffeine containing food or beverages for 5 hours prior to the study but will be allowed 8 ounces (one cup) of water during these 5 hours.

**DNA sampling** will be done using buccal cells obtained by swabbing the cheek area inside the mouth of the participant or by obtaining a saliva sample.

**Attention and memory testing**

To enhance participants’ attention and memory to displayed images, participants will be asked to remember the presented images and will be told that they will be quizzed on the images immediately after finishing a scan session. Testing will be performed immediately after participant has been removed from the scanner. As described by Killgore et al. (2003) the previously seen images will be mixed with 50% novel distractor images.

### Visits 2 and 6.

### Exercise.

### Exercise session will be performed during Visits #4 - #10 at random order.

1. Vigorous exercise.

Each participant will be asked to run/jog on the treadmill for up to 30 minutes at the speed that would be about 20-50% lower than the highest speed they were able to run during the treadmill test at Visit #1 or at a speed that is comfortable to the participant.

During the exercise session, heart rate will be monitored.

1. Light exercise.

Each participant will be asked to walk on the treadmill for 30 minutes at their casual walking speed (2.0 -2.5 METs – metabolic equivalents). During the exercise session, heart rate will be monitored.

**Urine Samples** –Participants will be supplied with urine collection cups and asked to collect a spot urine sample at the beginning of each visit before the exercise session starts.

**Scans**

Scans will be obtained after 30 minute rest following the exercise. We will conduct neuroimaging sessions at times that are convenient for the participants. The combination of weekday and weekend scan options is chosen to optimize participation of participants who may work or be otherwise occupied during weekdays.

**Stimuli**

Cannabis stimulus

Content: We will use cannabis stimulus as described by Field et al.(Field, Eastwood et al. 2006). It includes pictures of cannabis in different forms and its use (people smoking joints) and related paraphernalia (e.g. bongs, pipes).

Food stimulus.

Content: Existing studies have shown clear brain effects of food cues that activate limbic, cognitive and reward regions. (Holsen et al., 2005). Killgore et al. (2003) have shown that caloric content of the food stimulus employed (i.e. high calorie versus low calorie) produces differential effects on regional brain activation. In this study we will use a stimulus set that includes only high caloric (high energy) food cues versus nature comparisons.

Paradigm: A block trial design is planned based upon our desire to employ a previously successful paradigm (Killgore et al., 2003) and because our goal is to maximize our chances of detecting activation differences (Birn et al., 2002). We will provisionally model our design on that of Killgore et al., 2003 by employing individual scanner runs of food images, cannabis-related images, and nature scenes. To control for non-specific aspects of visual stimulation, we will used blurred Gaussian images (similar to Holsen et al., 2005) as the fixation task. After Killgore et al., (2003) we will present images in 30-second blocks of 10 images each, displayed for 2500 milliseconds with a 500 millisecond interstimulus delay. To minimize the risk of participant motion effects precluding trial analysis, we will keep limit each scanner run to 3 minutes 30 seconds (i.e. 3 target stimulus 30 second blocks, 4 blurred Gaussian background blocks) minutes. The particular food, cannabis, or nature image sequences used will be randomized across scans and the order will be counterbalanced. Participants will have 3 scanner runs of the 3 minute 30 second stimulus display per exercise condition, so that each stimulus set will be seen 3 times/exercise condition. A different set of images will be used for the moderate and vigorous interventions and will be balanced across participants to neutralize order effects.

The total time in the scanner is about 1 hour/day.

**Image Acquisition**

Collected images will include structural images for functional data overlay, diffusion tensor images for correlating fiber tract integrity with craving scores, resting functional images for baseline connectivity effects, and task-evoked functional images for examining image-induced regional brain activation. All images acquisitions do not involve contrast and are minimal risk. 128 T1 volumetric 3-D SPGR whole-brain sagittal images will be acquired from all study participants at 3 T with a SENSE head coil. Parameters: Flip = 20 degrees, TE = 4.2 ms, slice thickness = 1.2 mm, no skip, field of view (FOV) = 24x 24 cm, matrix size 256 x 256 pixels for an in-plane resolution = 0.88 mm. Whole brain 3-D images will be used for overlay of functional data in 3-D space. We will acquire whole-brain functional oblique slices of 4.5 mm thick covering the regions of interest (TE=35 ms, TR=2 s, Flip=790, matrix 64 x64. Images will be collected on the 3.0 T Philips scanner using a quadrature head coil. We will collect whole-brain axial slices of 4.5 mm thick with 0.5 mm skip. We will analyze fMRI data using the Brain Voyager QX software (Brain Innovation, Maastricht, The Netherlands) or other analysis package employing the general linear model (GLM) to construct statistical parametric maps SPMs to examine regional brain activation in response to photic stimulation and the semantic task. Preprocessing: 3-D motion correction (runs having over 2 mm translational motion or more than 1 degree rotational motion will be excluded), spatial smoothing (4 mm FWHM Gaussian kernel), temporal smoothing (linear trend removal and 3-Hz high pass filtering) (Skudlarski et al., 1999). Statistical threshold for activation will be a false discovery rate of 0.05, corrected for the number of multiple independent whole-brain comparisons. The stimulus paradigm time courses will be convolved with a hemodynamic response gamma function (Boynton et al., 1996). Following SPM construction, activation within a desired region of interest will be determined as the number of pixels reaching statistical threshold and the percent BOLD signal change within the activated pixel region. The method for choosing ROI's is defined below.

1. **Risks**

This study will include adults (18-35 years old).

Potential Risks of fMRI Imaging:

*Physical: -* Magnetic resonance procedures themselves do not utilize ionizing radiation, and there are no common physical risks to MRI procedures. Minor physical discomfort may occur while lying in the scanner.

*Psychological:* – Potential psychological risks include anxiety during various procedures. Some individuals (5-10%) become claustrophobic in the MR scanner, leading to premature termination of the study. Proposed measures to minimize imaging risks -- The Imaging Institute, including Dr. Gore’s group, has extensive experience in MRI research studies with adults. Our staff and imaging center personnel are very experienced in keeping participants comfortable during scanning procedures. Participants are carefully screened for indwelling metal or medical devices that might pose a danger in the scanner. We follow a complete and in-depth screening procedure designed to identify any participant with a mental health disorder that might make him/her particularly vulnerable to these procedures.

Potential Risk of Exercise Testing (VO_2max_)

To assess physical capability to perform exercise (walking and running), participant will be given a graded-treadmill test using age appropriate protocol. During the test, participant will be closely monitored and will be instructed to stop the test when they choose. The test will be stopped by the research team if their heart rate exceeds their maximum heart rate (220 – age). The test will be preceded with assessment performed by a physician or designated nurse and would include EKG to screen out participants with abnormal EKG response from the treadmill test.

The criteria include:

- ST-segment depression (defined as ≥1.0 mm of horizontal or down-sloping ST at 80 msec beyond the J point, suggesting myocardial ischemia)
- Ventricular arrhythmias, presenting as frequent multiform PVC, and especially in combination with ST depressions
- Heart rate response that >2 standard deviations (>20 beats/min) below the age-predicted maximal HR who are limited by volitional fatigue
- A change in Diastolic blood pressure >15 mmHg
- Anginal symptoms > 2 (out of 1-4 scale, corresponding to chest pains of perceptible but mild, moderate, moderately severe, and severe intensities, respectively).

Potential Risk of Exercise.

There is potential risk of injury caused by walking or jogging on the treadmill. The common symptoms include shortness of breath, or more severe, such as falling down. Also, there may be the rare instance of heart problems, such as a heart attack, that could be life threatening. We will do a fitness test to screen if each participant is capable to walk or run for 20 to 30 minutes.

Potential Risks of Genotyping.

There is a very low risk that genotyping information may be disclosed and participant could suffer potential embarrassment or economic harm.

Potential Social/Legal Risks.

There are potential social risks to participants if the confidentiality of the information they provide us is disclosed. For this reason, we follow strict confidentiality procedures.

1. **Reporting of Adverse Events or Unanticipated Problems involving Risk to Participants or Others**

An adverse event (AE) is any untoward medical occurrence in a participant, not necessarily having a causal relationship with the study. A serious adverse event (SAE) is any untoward medical occurrence that a) results in death, b) is life-threatening, c) requires inpatient hospitalization or prolongation of existing hospitalization, d) results in persistent or significant disability/incapacity, or e) is a congenital anomaly/birth defect.

AE’s are graded as:

- Mild (no limitation of usual activities)
- Moderate (some limitation)
- Severe (inability to carry out usual activities)

AE’s are attributed according to the relationship to the study procedures as:

- Not related
- Unlikely
- Possible
- Probable
- Definite

SAE’s are reported immediately by telephone to the VU IRB. Adverse events will be detected by clinical examinations by the studies clinical investigator. An Adverse Event Report form will be completed and returned to the VU IRB within 5 working days. Summary Reports are submitted to the VU IRB and will contain a) The number of adverse events and an explanation of how each event was handled, b) The number of complaints and how each complaint was handled, c) The number of participant withdrawals and an explanation of why the participant withdrew or was withdrawn, and d) The number of protocol violations and how each was handled.

1. **Study Withdrawal/Discontinuation**

Participants will be withdrawn from the study for safety considerations, non-compliance with study procedures, difficulties with scheduling or participation, excess movement or anxiety during scanning, or other reasons that the PI or co-investigators conclude will impair the scientific utility of the study. Participants will be provided an explanation if they are withdrawn from the study by the investigators.

1. **Statistical Considerations**

**General statistical methods**

Percent BOLD signal change in the targeted ROIs, as well as demographic characteristics of the experimental and control group will be summarized using descriptive statistics. Given proposed group sizes of 10 participants (with no missing data), we expect to use parametric statistical analysis procedures. However, samples of this size are highly sensitive to outlier values, thus visualization and statistical investigations of parametric assumptions will be conducted. Because relative (greater or lesser) differences in activation rather than absolute values of those differences are of primary interest in this research, if distributions do not meet parametric assumptions, depending on the nature of the violations methods based on ranks will be used or outliers will be dealt with on a case-by-case basis.

Assuming multivariate normality and homogeneity assumptions are met, multivariate profile analysis (Tabachnick & Fidell, 2000) will be used to investigate differential patterns of ROI activation (profiles) between the two primary study groups of cannabis users and non-user controls (Primary Specific Aim 1). A statistically significant interaction effect of study group and ROI (e.g., insula, inferior frontal cortex, ventrolateral prefrontal cortex, parahippocampal gyrus, bilateral fusiform gyrus) will indicate statistically significant differences in activation (% BOLD signal change) profiles. Given a statistically significant interaction effect from an overall profile analysis, post-hoc tests controlling the experiment-wise Type I error rate will be used to determine precisely which component members of the profile (i.e., areas of activation or ROIs) are contributing to the overall profile difference, as well as the magnitude and direction of the specific component difference. These effect sizes will be used for statistical powering estimates in the planning of subsequent RO1 research.

Graphical and descriptive statistical techniques will be used to explore patterns of changes in activation within subjects to cannabis cues after the two exercise conditions (Primary Specific Aim 2). Tests of the changes in activation within key ROIs will be conducted using general linear modeling (GLM) methods that incorporate Generalized Estimation Equations (GEE) for parameter estimation of correlated data. The combination of GLM and GEE provides the most flexibility for modeling data of uncertain distribution, as well as for incorporating the mixed effects of between (cannabis vs. control) and within (prior exercise state). Again, effect sizes generated from these analyses will be used for statistical powering of planned subsequent RO1 research.

**Power Estimates**

Because this is a pilot study to demonstrate feasibility, we estimate 10 subjects per group as reasonable sample size as this group size is commonly well in fMRI pilot studies. A key outcome of this study will be the calculation of effect sizes that will inform the powering of subsequent, larger studies. Commercially available statistical software packages (NCSS and PASS, number Cruncher Statistical Systems, Kaysville, Utah; SPSS, Chicago, IL; Stata, College Station, TX) will be used for data analysis.

1. **Privacy/Confidentiality Issues**

The participant research file will contain information concerning mental and physical health and experimentally derived data. All data collected in this study will be used specifically for research purposes. Should information that might have a bearing on the well being of participants be discovered, this information will be communicated to participants and, at the participant’s request, with their physician.

Electronic databases containing identifiable participant information will be password encoded. Written information containing participant identifiers (informed consent, lab results, participant payment, etc) will be stored in file cabinets in offices with the departments of Radiology and Psychiatry. Participants will be assigned an alphanumeric code that will be used to label all research data.

1. **Follow-up and Record Retention**

This pilot study is expected to last one year with data analysis continuing indefinitely. Records may be destroyed after data analysis is complete and all manuscripts are published, but records may be maintained indefinitely for future analysis. Emerging methods of analysis in fMRI research may permit the reanalysis of previously acquired data to extract new meaning.

All information will be kept on computer storage disks or as paper for at least a 5-year period of time (including 5 years following the completion of these studies for data analysis). The computing centers of Vanderbilt University's Imaging Institute and the Division of Adult Psychiatry will be used to store and analyze the MRI data. Records will be coded by assigning an alphanumeric (not their initials) code for all documentation and data analysis sheets. The principal investigator, co-investigators, and research assistants working on the study will have access to the information. Identifying data such as name, address, etc. will be stored on computer using password-protection encryption and in locked files when in written format.

If paper records are to be destroyed those containing participant identifiers will be shredded directly or transferred to the hospital’s shredding service. If electronic data containing participant identifiers is to be destroyed, it will be disposed of using a medium-appropriate destruction method to prevent recovery. Data not containing participant identifiers will be disposed of by any convenient method.
