# Supplementary material for: Aerobic Exercise Training Reduces Cannabis Craving and Use in Non-Treatment Seeking Cannabis-Dependent Adults
Source: PLoS One. 2011 Mar 8;6(3):e17465. doi: 10.1371/journal.pone.0017465 (PMC3050879; doi:10.1371/journal.pone.0017465)
Supplement: Consent Form S1 — (DOC) [file pone.0017465.s003.doc]

This informed consent document applies to 18-35 years old

Name of participant: _____________________________________________Age: _____

The following information is provided to inform you about the research project and your participation in it. Please read this form carefully and feel free to ask any questions you may have about this study and the information given below. You will be given an opportunity to ask questions, and your questions will be answered. Also, you will be given a copy of this consent form.

Your taking part in this research study is voluntary. You may choose not to take part without affecting your healthcare/services or other rights. You are also free to withdraw from this study at any time. In the event new information becomes available that may affect the risks or benefits of this research study or your willingness to participate in it, you will be told so that you can make an informed decision whether or not to continue being in this study.

1. **What is the purpose of this study?**

You are being asked to take part in a research study because you are currently dependent on cannabis (marijuana). The purpose of this study is to find out how exercise (physical activity) may affect your brain response to pictures of foods and pictures showing cannabis and people smoking cannabis.

About thirty-five people will take part in this study.

1. **What will happen and how long will you be in the study?**

**Study Visit #1**

If you decide to take part in this study, you will come to the Clinical Research Center (CRC) at Vanderbilt University. Before the first study visit, the study team will have spoken to you on the phone to get information about scheduling a visit at the CRC (e.g. address, birth date, contact information, etc). When you arrive at the CRC we will go over the consent form and study information again. Table 1 has the complete list of tests and when they are to be done during each visit. You may be asked to come to one or more (up to twelve) visits. Visits #1, #2, and #3 will take about 2- 3 hours will be scheduled 2-7 days apart. Visits #4 to #12 will take 30-60 minutes and will only include jogging or walking on a treadmill.

Table 1. Visit Schedule

| **Procedure** | **Visit 1**  **(day 1)** | **Visit 2**  **(day 2-8)** | **Visit 3**  **(days 5-14)** | **Visit 4-12**  **(days 2-10)** |
| --- | --- | --- | --- | --- |
| Consent | X |  |  |  |
| Surveys/questionnaires***** | X | X | X | X |
| Anthropometrics and Vital Signs | X |  |  |  |
| EKG (heart function test) | X |  |  |  |
| Fitness test* | X | X | X |  |
| Exercise** |  | X | X | X |
| fMRI*** | X | X | X |  |
| Urine Tests | X | X | X | X |
| Buccal cells (DNA)**** | X |  |  |  |
| Breath test | X | X | X |  |

* assessment of physical fitness. This test will be done during Visit #1, #2, or #3.

** exercise in randomized order. You will be asked to run or jog on a treadmill at a speed approximately 20-50% lower than your fast running speed and/or walk at a comfortable speed during Visit #4 – Visit #12.

*** functional magnetic resonance imaging (test to picture parts of your brain). This will be done twice, either during Visit #1 and Visit #3 or during Visit # 2 and Visit #3.

**** saliva sample

***** on the days you are not scheduled for a visit, we will call you and ask you questions from the Marijuana Craving Questionnaire, Caffeine and Nicotine Use questionnaire, and Drug use (question Page 13 Question 1: Marijuana (as a dried plant). You will get copies of these questionnaires so you would know questions we will be asking.

1. Vital signs and anthropometrics (measure of size and shape). – We will measure your weight, height, blood pressure, heart rate, and body temperature.

2. Surveys and questionnaires. We would like to complete 5 questionnaires asking you about your mood, behaviors, use of nicotine, caffeine, alcohol, and some recreational drugs. Some of these questionnaires will be repeated during the study and done over the telephone as specified in the schedule table. If you would not be available, you would be able to call back and leave a message and/or information.

3. Fitness test – We will measure how physically fit you are. You will have an EKG (a test showing how your heart functions) before doing the treadmill test. This test does not cause pain; however, it is possible that sticky pads used for the EKG may irritate your skin. You will be asked to walk and run for about 20 minutes on a treadmill. The treadmill will go a little faster or a little steeper (like going up a hill) every few minutes. During this test, we will study the amount of oxygen, the air breathed in, and the amount of carbon dioxide, the air you breathed out. Your heart rate will be watched and a doctor will be at the Clinical Research Center to help in the case of an emergency. A nurse and research person will be in the room during the fitness test. When you feel you are too tired and are not able to continue the test, or if you feel any chest pain or discomfort, the test will be stopped.

4. Urine testing. You will give us a small (half a cup) urine sample when you come to the Clinical Research Center. We will test for pregnancy (women of childbearing potential only) and for use of recreational drugs.

1. Breath testing. We will ask you to blow at the testing device to check if there is alcohol present in your breath.
2. Buccal DNA – We may ask you to rinse mouth with a small glass of mouth rinse (Scope) and spit it into a plastic container. The purpose of its collection is explained at the end of the consent form.
3. Fasting – we will ask you to avoid eating any food or caffeine containing beverages for at least 4 hours before the study.

Visit #2 and Visit #3.

Visits #2 and #3 will be scheduled at 2-7days apart. Each visit will consist of filling out surveys, urine tests, exercise test (running or walking), and fMRI scan. You will be asked not to eat for 4 hours before coming to the study visit and to avoid alcohol for at least 24 hours before coming to the study.

1. Running on a treadmill. We will ask you to run up to 30 minutes on a treadmill at the speed that would be about 20-50% lower than the highest speed you were able to run at the treadmill test during Visit #1 or that is comfortable to you. You will be able to stop the test if you will feel tired or if you feel any chest pain or discomfort.
2. Running or walking on treadmill (Visit #4 – Visit #10).

We may be asked you to come 2-10 times to run or walk on a treadmill between days 2-14.

1. Walking on a treadmill.

We will ask you to walk slowly on a treadmill at the speed that is comfortable to you.

4. Functional magnetic resonance imaging (fMRI) scan.

Before or after exercise and after 20-30 minutes rest, you will go to another part of the medical center for a test called functional magnetic resonance imaging. The fMRI uses a computer to make pictures and measure your brain. The fMRI scan will take about 30 minutes and is taken in a large machine that is shaped like a tunnel. This scan does not use x-rays. Instead, the scan uses a strong magnet and radio waves, like those used in an AM/FM radio to make pictures of your body.

You may not be able to have this scan if you have a device in your body, such as aneurysm clips in the brain, heart pacemakers or defibrillators, and cochlear (inner ear) implants. Also, you may not be able to have this scan if you have iron-based tattoos or pieces of metal (bullet, BB, shrapnel) close to or in an important organ (such as the eye).

Certain metal objects like watches, credit cards, hairpins, writing pens, etc. may be damaged by the machine or may be pulled away from the body when you are getting the scan. Also, metal can sometimes cause getting poor pictures if it is close to the part of the body being scanned. For these reasons, you will be asked to remove these objects before going into the room for the scan.

You will hear “hammering”, clicking, or squealing noises during the scan. You will be given earplugs to reduce the noise. You will also be told how to alert the staff if you need them.

During the scan, the fMRI staff is able to hear and talk to you.  You will also be able to hear the staff.  They will be talking to you during your scan and may ask you to hold your breath, not move, or other simple tasks. You may be asked to lie very still throughout the scan. During the scans, you will look at pictures, including some pictures of food, cannabis, and people smoking cannabis. The fMRI will show us what part of your brain is working while you look at the pictures. After each fMRI scan, we will ask you some questions about what you saw, and how you feel after looking at these pictures.

In this study, the fMRI scan is for research only. But, if we see something that is not normal, you will be told and asked to consult your doctor.

**All procedures are being done for research purposes.**

1. **Costs to you if you take part in this study:**

There will be no costs to you or your insurance company as a result of your taking part in this study. All of the tests done in this study are for research reasons only. You and/or your insurance company will not be responsible for any costs linked to your being a part of this study.

**4. Side effects and risks that you can expect if you take part in this study:**

None of the tests are harmful. Overall, the risks are low. All studies will be done by trained persons, including the research doctors and their assistants and nurses.

**Health and drugs, alcohol, and nicotine use questions:**

Some questions may be hard or make you uneasy to answer.

**fMRI Scan:**

There are no known major risks with an fMRI scan. But, it is possible that harmful effects could be found out in the future. Even though the tunnel is open, it may bother you to be placed in a tight space (claustrophobia), and to hear the noise made by the magnet during the scan. You will be given earplugs to reduce the noise. You may also feel the table vibrate and/or move slightly during the scan. It may be hard to lie on the table during the scan. If you have any metal pieces in your body, they could move during the scan and damage nearby tissues or organs. There are no known risks of having fMRI scans without contrast while pregnant.  However, there may be risks that are unknown.

**Fitness Test**

While we do not expect any problems with this testing, you may have shortness of breath and feel tired which is common. Should you have chest pain or any other problems, which could be severe, testing will be stopped. You may also run the risk of falling down while exercising, which could cause injury. There are side handrails on the treadmill and a stop button for your safety.

The study procedures may hurt an unborn child. If you take part in this study must use two forms of birth control as described above. If you become pregnant while you are in this study, you must tell your doctor at once. Also, women must not breast feed while in this study. If you are a woman and are able to become pregnant, you will have blood and urine tests to make sure that you are not pregnant while in this study.

**5.** **Risks that are now known:**

Although we do not expect you have any risks for being in this study, there may be unknown or unforeseeable risks.

**6.** **Payment in case you are injured while in this study:**

If it is determined by Vanderbilt and the Investigator that an injury occurred as a direct result of the tests or treatments that are done for research, then you and/or your insurance will not have to pay for the cost of immediate medical care provided at Vanderbilt to treat the injury. There are no plans for Vanderbilt to pay for the costs of any additional care. There are no plans for Vanderbilt to give you money for the injury.

1. **Good effects that might result from this study:**

a) The potential benefits to science and humankind that may result from this study are:

This study will increase our understanding of how exercise could affect brain function in cannabis users.

b) The potential benefits to you from this study are:

There are no benefits to you from taking part in this study.

1. **Other treatments you could get if you decide not to be in this study:**

This is not a treatment study. You may decide not to take part in this study.

1. **Payments for your time spent taking part in this study or expenses:**

To compensate you for your time and effort, you will be paid for coming in for the study procedures. You will be paid $50 for Visit #1. You will be paid maximum of $250 for coming and completing all tests (including fMRI) required in Visit #2 and Visit #3 or up to $300 for completing visits 4-12.

1. **Reasons why the study doctor may take you out of this study:**

You may be withdrawn if the investigators have concerns about your safety or for other reasons such as trouble setting up your appointments. You will be taken out of the study if you have a positive drug other than cannabis (for cannabis users) or pregnancy screens. If you are taken out of the study, you will be told why.

You may be withdrawn from the study by the research doctor if there is: a change in your health status or ability to perform study tests or if you wish to stop being in the study. If you are withdrawn from the study, you will be told the reason.

1. **What will happen if you decide to stop being in this study?:**

If you decide to stop being part of the study, you should tell this the study staff. Deciding not to be part of the study will not change your regular medical care in anyway.

**12. Who to call for any questions or in case you are injured:**

If you should have any questions about this research study or if you feel you have been hurt by being a part of this study, please feel free to contact the Principal Investigator, Mac Buchowski, Ph.D. at 343-4192, or the study doctor, Dr. Ronald Cowan, at (615) 322-2303.

For additional information about giving consent or your rights as a person in this study, please feel free to call the Vanderbilt University Institutional Review Board Office at (615) 322-2918 or toll free at (866) 224-8273.

1. **Confidentiality:**

All efforts, within reason, will be made to keep the personal information in your research record private but total privacy cannot be promised. Your information may be shared with Vanderbilt or the government, such as the Vanderbilt University Institutional Review Board, Federal Government Office for Human Research Protections, and National Institutes of Health.

To help us protect your privacy, we have obtained a Certificate of Confidentiality from the National Institutes of Health. With this Certificate, the researchers cannot be forced to disclose information that may identify you, even by a court subpoena, in any federal, state, or local civil, criminal, administrative, legislative, or other proceedings. The researchers will use the Certificate to resist any demands for information that would identify you, except as explained below.

The Certificate cannot be used to resist a demand for information from personnel of the United States Government that is used for auditing or evaluation of federally funded projects or for information that must be disclosed in order to meet the requirements of the federal Food and Drug Administration (FDA).

You should understand that a Certificate of Confidentiality does not prevent you or a member of your family from voluntarily releasing information about yourself or your involvement in this research. If an insurer, employer, or other person obtains your written consent to receive research information, then the researchers may not use the Certificate to withhold that information.

The Certificate of Confidentiality does not prevent the researchers from disclosing voluntarily, without your consent, information that would identify you as a participant in the research project under the following circumstances: if you or someone else is in danger.

1. **Authorization to Use/Disclose Protected Health Information:**

All efforts, within reason, will be made to keep your protected health information (PHI) private. PHI is your health information that is, or has been gathered or kept by Vanderbilt as a result of your healthcare. This includes data gathered for research studies that can be traced back to you. Using or sharing (“disclosure”) such data must follow federal privacy rules. By signing the consent for this study, you are agreeing (“authorization”) to the uses and likely sharing of your PHI. If you decide to be in this research study, you are also agreeing to let the study team use and share your PHI as described below.

As part of the study, Dr. Buchowski and his study team may share the results of your study and/or non-study linked laboratory tests and magnetic resonance imaging data, as well as parts of your medical record, to the groups named below. These groups may include people from the Federal Government Office for Human Research Protections, the Vanderbilt University Institutional Review Board*.*  Federal privacy rules may not apply to these groups; they have their own rules and codes to assure that all efforts, within reason, will be made to keep your PHI private. The sponsor may give your health data, without your name, to others or use it for other research projects not listed in this form. The sponsor, Vanderbilt, Dr. Buchowski and his staff will comply with any and all laws regarding the privacy of such information.

The study results will be kept in your research record for at least six years after the study is finished.  At that time, the research data that has not been put in your medical record will be destroyed*.* Any research data that has been put into your medical record will be kept for an unknown length of time.

Unless told otherwise, your consent to use or share your PHI does not expire. If you change your mind, we ask that you contact Dr. Buchowski, PhD and let him know that you withdraw your consent.  His contact address is 1161 21^st^ Ave South, Medical Center North, Room A-4013.  At that time, we will stop getting any more data about you.  But, the health data we stored before you withdrew your consent may still be used for reporting and research quality.

If you decide not to take part in this research study, it will not affect your treatment, payment or enrollment in any health plans or affect your ability to get benefits. You will get a copy of this form after it is signed.

**STATEMENT BY PERSON AGREEING TO PARTICIPATE IN THIS STUDY**

**I have read this informed consent document and the material contained in it has been explained to me verbally. All my questions have been answered, and I freely and voluntarily choose to participate.**

**Do you agree to complete additional/optional questionnaires?**

**YES NO (circle one)**

Date Signature of patient/volunteer

Consent obtained by:

Date Signature

Printed Name and Title

# Consent for Genetic Research

The purpose of this study is to look at genes (DNA) and how they affect health and disease. Genes are the instruction manual for your body. The genes you get from your parents decide what you look like and how your body behaves. They can also tell us a person’s risk for certain diseases and how they will respond to treatment.

You are being asked to give a saliva sample for genetic research. What we learn about you from this sample will not be put in your health record. Your test results will not be shared with you or your doctor. No one else (like a relative, boss, or insurance company) will be given your test results. Your sample will only be used for research at Vanderbilt University and will not be sold.

One risk of giving samples for this research may be the release of your name that could link you to the stored samples and/or the results of the tests run on your samples. This may cause problems with insurance or getting a job. To prevent this, these samples will be given a code. Only the study staff will know the code. The name that belongs to the code will be kept in a locked file or in a computer with a password. Only Dr. Buchowski will have access to your name.

Your samples will be used for research only and will not be sold or used to make products that could be sold for money. Your samples and information about you may be shared with others to use for research. To protect your privacy, we will not release your name.

You will not receive any benefit as a result of the tests done on your samples. These tests may help us learn more about the causes, risks, treatments, or how to prevent health problems. Giving samples for research is your free choice and you may be in the study even if you do not want your samples used or stored for gene research.

At any time, you may ask to have your sample destroyed. You should contact Dr. Buchowski at 615-343-4192 to have your sample destroyed and no longer used for research. We will not be able to destroy research data that has already been gathered using your sample. Also, if your identity was removed from the samples, we will not be able to locate and destroy them.

There will be no costs to you for any of the tests done on your samples. You will not be paid for the use of your samples.

Please check Yes or No to the questions below:

My blood/tissue sample may be used for gene research.

Yes  No

My blood/tissue sample may be stored/shared for future gene research in addiction or physical exercise.

Yes  No

My blood/tissue sample may be stored/shared for future gene research for other health problems (such as cancer, heart disease, etc).

Yes  No

Signature: ______________________________________Date:___________
