# Supplementary material for: Aerobic Exercise Training Reduces Cannabis Craving and Use in Non-Treatment Seeking Cannabis-Dependent Adults
Source: PLoS One. 2011 Mar 8;6(3):e17465. doi: 10.1371/journal.pone.0017465 (PMC3050879; doi:10.1371/journal.pone.0017465)
Supplement: Approval Letter S1 — (PDF) [file pone.0017465.s004.pdf]

Vanderbilt University
Institutional Review Board
504 Oxford House Nashville, Tennessee 37232-4315
(615) 322-2918 Fax: (615) 343-2648
www.mc.vanderbilt.edu/irb

May 21, 2010

Maciej S. Buchowski, Ph.D.
Medicine - Gastroenterology
A-4103 MCN 2195

Natalie Meade
Gastroenterology
A-4103 MCN

RE: IRB# 081302 "Interactions between Physical Activity and Cannabis Use in Adults"

Dear Maciej S. Buchowski, Ph.D.:

A sub-committee of the Institutional Review Board reviewed the amendment dated 5/10/2010 for the research study identified above. The sub-committee determined the changes to the study pose no additional risk to participants.

Amendment Description: This amendment changes the number of participants to 35 and changes the number of visits to up to twelve. This amendment also changes the compensation for visits 4-12 where participants can receive up to $300. Those changes are reflected in the IRB Application, the Consent Forms Cases and Controls dated 5/10/2010 and in the Protocol submitted on 4/30/2010.

The Consent Form(s) have been stamped with the approval and expiration date and this copy should be used when obtaining the participant's signature. Federal regulations require that the original copy of the participant's consent be maintained in the principal investigator's files and that a copy be given to the subject at the time of consent. An additional record (i.e., case report form, medical record, database, etc.) of the consent process should also be maintained in a separate location for documentation purposes.

As the Principal Investigator, you are responsible for the accurate documentation, investigation and follow-up of all possible study-related adverse events and unanticipated problems involving risks to participants or others. The IRB Adverse Event reporting policy III.G is located on the IRB website at http://www.mc.vanderbilt.edu/irb/.

Any further changes to the study must be presented to the IRB for approval prior to implementation.

DATE OF AMENDMENT APPROVAL: May 21, 2010

Sincerely,

James B. Atkinson, M.D., Ph.D., Vice-Chair
Institutional Review Board
Health Sciences Committee #1

JBA/adh

Electronic Signature: James Atkinson/VUMC/Vanderbilt : (53F618306DD90C220C0ACBE60D90BF7E)

Signed On: 05/22/2010 04:34:40 PM CDT
